# An Iterative Pseudo Label Generation framework for semi-supervised hyperspectral image classification using the Segment Anything Model

**DOI:** 10.3389/fpls.2024.1515403

**Published:** 2024-12-23

**Authors:** Zheng Zhao, Guangyao Zhou, Qixiong Wang, Jiaqi Feng, Hongxiang Jiang, Guangyun Zhang, Yu Zhang

**Affiliations:** ^1^ School of Astronautics, Beihang University, Beijing, China; ^2^ Aerospace Information Research Institute, Chinese Academy of Sciences, Beijing, China; ^3^ Center of Remote Sensing, Nanjing Tech University, Nanjing, China

**Keywords:** hyperspectral image classification, remote sensing, semi-supervised learning, Segment Anything Model, pseudo label generation

## Abstract

Hyperspectral image classification in remote sensing often encounters challenges due to limited annotated data. Semi-supervised learning methods present a promising solution. However, their performance is heavily influenced by the quality of pseudo labels. This limitation is particularly pronounced during the early stages of training, when the model lacks adequate prior knowledge. In this paper, we propose an Iterative Pseudo Label Generation (IPG) framework based on the Segment Anything Model (SAM) to harness structural prior information for semi-supervised hyperspectral image classification. We begin by using a small number of annotated labels as SAM point prompts to generate initial segmentation masks. Next, we introduce a spectral voting strategy that aggregates segmentation masks from multiple spectral bands into a unified mask. To ensure the reliability of pseudo labels, we design a spatial-information-consistency-driven loss function that optimizes IPG to adaptively select the most dependable pseudo labels from the unified mask. These selected pseudo labels serve as iterative point prompts for SAM. Following a suitable number of iterations, the resultant pseudo labels can be employed to enrich the training data for the classification model. Experiments conducted on the Indian Pines and Pavia University datasets demonstrate that even a simple 2D CNN based classification model trained with our generated pseudo labels significantly outperforms eight state-of-the-art hyperspectral image classification methods.

## 1 Introduction

With the continuous advancement of remote sensing technology, hyperspectral imagery has garnered increasing attention due to its rich spectral information. For instance, Mérida-García et al. (2024) analyzed wheat gene dissection using hyperspectral images, and Nagy et al. (2024) employed machine learning algorithms to estimate maize chlorophyll content. Hyperspectral image classification presents critical challenges and remains a fundamental component for the effective application of hyperspectral technology. Despite significant progress in deep learning, hyperspectral image classification techniques often rely heavily on extensive pixel-level annotations, which are both time-consuming and labor-intensive to obtain. To address these challenges, recent studies have proposed various methods to enhance the quality of hyperspectral data. For example, Han et al. (2024) addressed the issue of incomplete spectral coverage by utilizing spectral libraries to improve spectral resolution, effectively enriching spectral information from low-resolution or incomplete data. Additionally, Wu et al. (2024) introduced a novel network for super-resolution tasks, focusing on multi-scale background feature enhancement, enabling the effective recovery of high-resolution remote sensing images from low-resolution inputs. These advances have significantly improved the accuracy and reliability of hyperspectral image classification. However, they still fail to address the inherent challenge of limited labeled data. To tackle this issue, semi-supervised learning has emerged as a powerful approach in hyperspectral image classification, leveraging the combination of a small set of labeled data and a large amount of unlabeled data to optimize model’s performance.

Current semi-supervised learning methods for hyperspectral image classification typically generate pseudo labels from unlabeled pixels during training, which are then integrated into the classification network as additional training samples. A prevalent approach is the self-training scheme (Marconcini et al., 2009), which generates highly confident predictions to augment the available labeled data. Furthermore, Zhang et al. (2014) adapted a co-training process that incorporates both original spectral signatures and 2D Gabor features for classification in scenarios with extremely limited labeled samples. Haut et al. (2018) introduced a Bayesian CNN framework assisted by active learning for semi-supervised hyperspectral image classification, which iteratively strengthens the small set of labeled samples by selecting and annotating the most informative unlabeled data. Although semi-supervised learning approaches can increase the classification accuracy through generating pseudo labels, the credibility of these labels is often compromised due to the lack of prior knowledge in the early stages, leading to noise that negatively degrades the classification network’s performance.

In recent years, large language models have made remarkable advancements in natural language processing. Notably, models like GPT-3 (Patel et al., 2023), with billions of parameters, have demonstrated impressive capabilities in zero/few-shot learning. In the realm of computer vision, pre-trained visionlanguage models such as LLaVa (Liu et al., 2024) have exhibited exceptional zero-shot generalization performance across various visual tasks. Additionally, the Segment Anything Model (SAM) (Kirillov et al., 2023) showcases the ability to perform category-agnostic segmentation by utilizing visual cues such as boxes, points, or masks. Recent research has focused on distillation (Julka and Granitzer, 2023) techniques tailored for specific task scenarios and developing adapters (Huang et al., 2024), underscoring SAM’s adaptability and potential for customization. However, due to the spectral band specificity inherent in hyperspectral images, these large vision models often struggle to achieve satisfactory results when directly applied to hyperspectral image classification.

**Figure 1:**
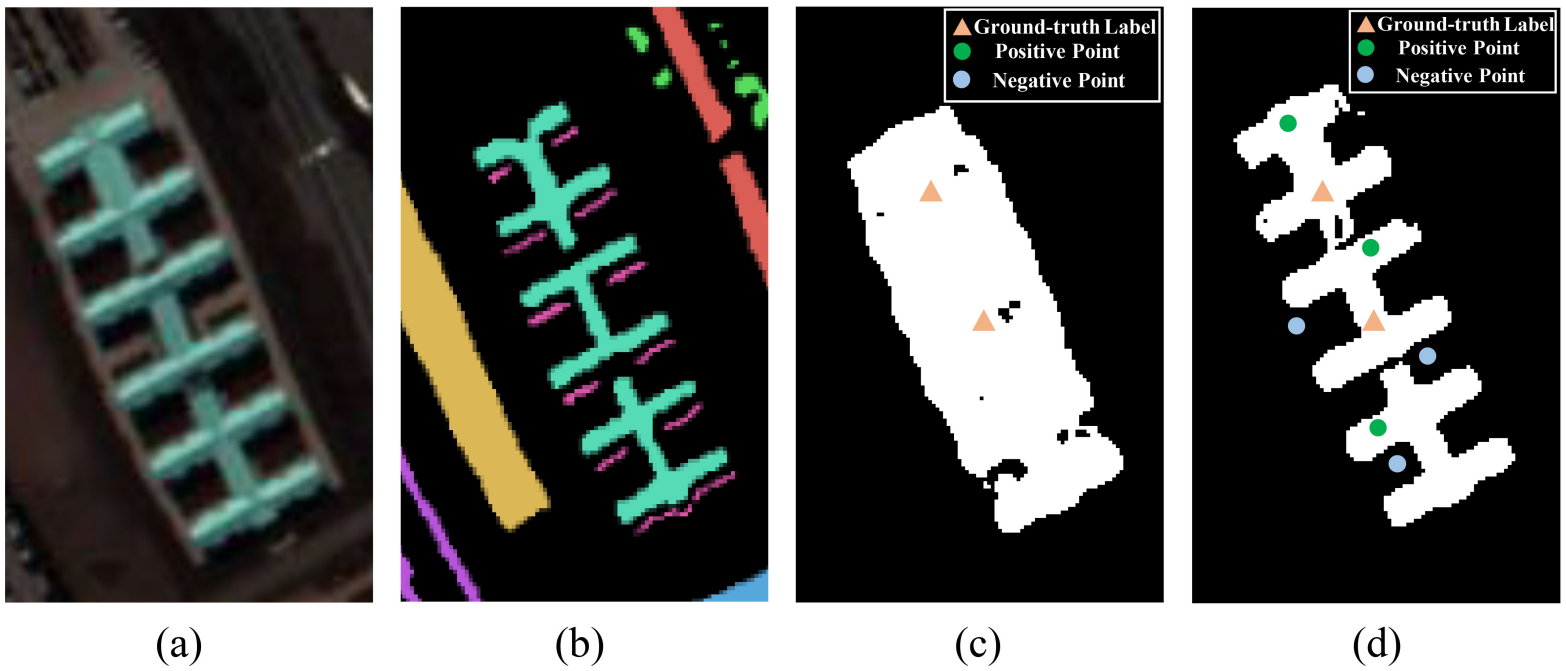
Illustration of the iterative generation process of pseudo labels by using point prompts to fine-tune SAM. **(A)** Hyperspectral image overlaid with the ground-truth label for a single object category, **(B)** Ground-truth labels, **(C)** Initial mask generated by SAM, **(D)** Mask generated by SAM after sufficient rounds of optimization with iteratively updated point prompts. A comparison between **(C, D)** demonstrates that refining the pseudo labels significantly enhances SAM’s prediction accuracy.

In this paper, we propose an Iterative Pseudo Label Generation (IPG) framework based on SAM to produce high-confidence pseudo labels. The iterative refinement of mask predictions for a given category is illustrated in **Figure 1**, demonstrating a notable increase in the confidence of predicted labels as input constraints become more accurate. Specifically, the spectral bands are divided into multiple groups, with every three adjacent bands forming a group, which are then input into the SAM model. A small number of annotated labels are used as point prompts for SAM to generate initial segmentation masks. To enhance accuracy, we introduce a spectral voting strategy to merge segmentation masks generated from multiple groups of spectral bands into a unified mask. Furthermore, a spatial-information-consistency-driven loss function is employed to optimize the IPG framework, enabling the dynamic generation of reliable pseudo labels from the unified mask. These pseudo labels iteratively serve as point prompts for SAM, with the final pseudo labels utilized for hyperspectral image classification.

The main contributions of this study are outlined as follows:

- We propose a SAM-based Iterative Pseudo Label Generation (IPG) framework for semi-supervised hyperspectral image classification, utilizing the structural prior knowledge of large models to generate reliable pseudo labels.
- To align the spectral channels of hyperspectral images with the input requirements of the SAM model, we divide the spectral bands into multiple groups, each comprising three adjacent bands. A spectral voting strategy is then employed to merge the segmentation masks generated from these groups into a unified representation, facilitating precise pixel-level classification.
- To enhance the reliability of pseudo labels derived from SAM segmentation masks, we develop a spatial-information-consistency-driven loss function. This function minimizes the feature distance between the generated pseudo labels and annotated labels in the spatial dimension, ensuring higher consistency and accuracy.

## 2 Proposed method

The proposed Iterative Pseudo Label Generation (IPG) framework leverages the Segment Anything Model (SAM) to iteratively refine pseudo labels for hyperspectral image classification. The process begins by decomposing hyperspectral images into a series of three-channel spectral bands. A limited set of annotated labels is then used to create initial point prompts for SAM, which generates segmentation masks for these spectral bands. These masks are aggregated using a spectral voting strategy to improve the pseudo labels’ reliability. Subsequently, a spatial-information-consistency-driven loss function is applied to identify high-confidence pseudo labels. These refined pseudo labels are iteratively fed back as new point prompts, enabling SAM to produce increasingly accurate labels with each iteration.

**Figure 2:**
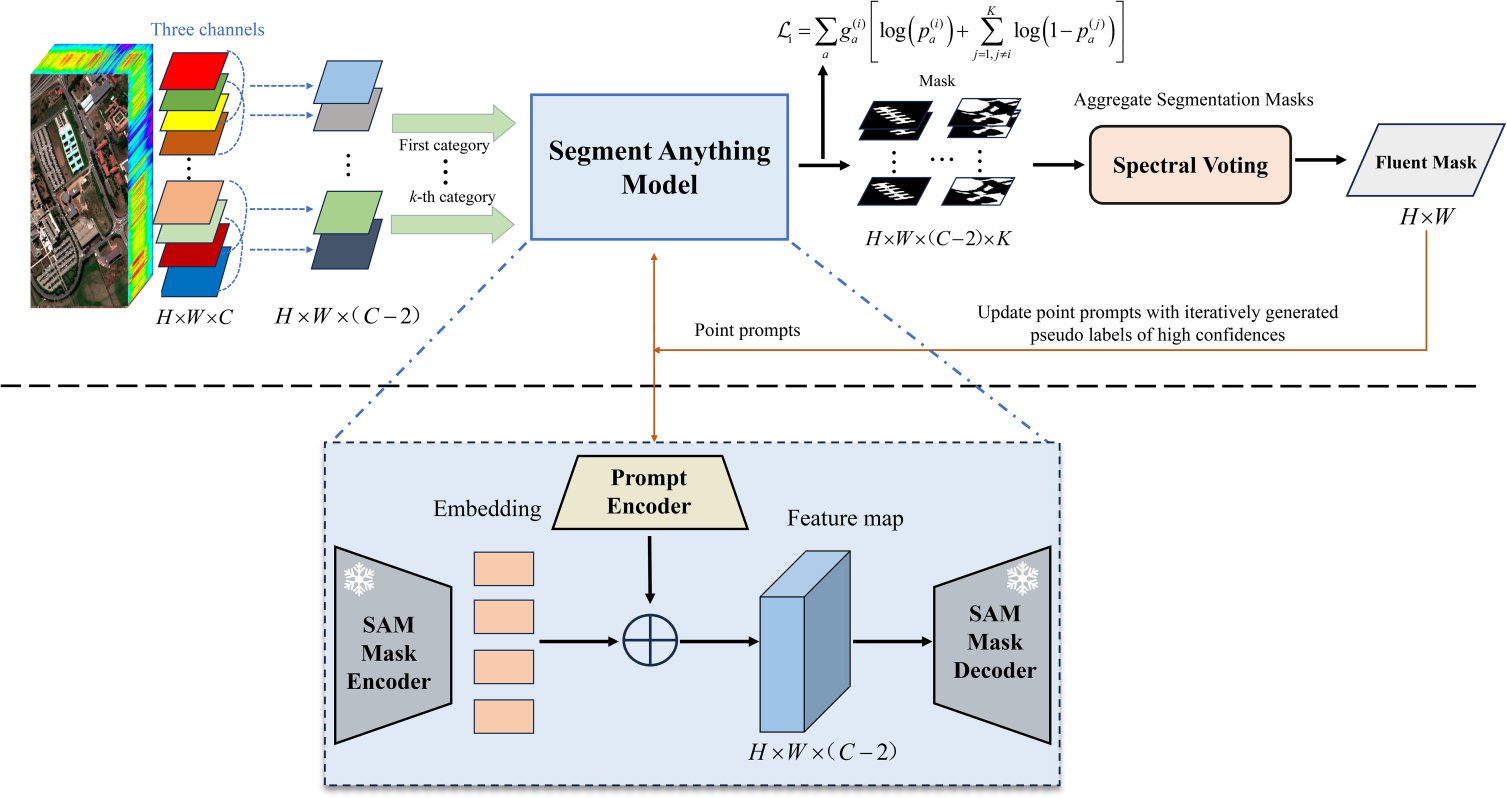
Illustration of the structure of the IPG framework. It introduces spectral voting strategy and spatial-information-consistency-driven loss function, freezing the SAM mask encoder and decoder, and updating prompt encoder, iteratively optimizing the generated pseudo labels.

For clarity, an overview of the IPG framework is presented in **Figure 2**. In the context of hyperspectral image classification, the classification model’s performance can be significantly enhanced through data augmentation using the pseudo labels generated by our IPG framework. Specifically, we concatenate these pseudo labels with the hyperspectral images, effectively expanding the training dataset and enriching the supervision information available for model training.

The following subsections provide an in-depth exploration of the IPG framework’s three core components (i.e., pseudo label generation, spectral voting, and consistency-driven loss function), and describe its application in hyperspectral image classification.

### 2.1 SAM-based iterative pseudo label generation

At the beginning, the input hyperspectral image is decomposed into a series of three-channel images along the channel dimension, enabling the integration of this hyperspectral image into the SAM image encoder module for segmentation purposes. To be specific, denote the input image by $M\in {\mathbb{R}}^{H\times W\times C}$ , which is decomposed along the channel dimension, establishing a sequence of three-channel images $Q\in {\mathbb{R}}^{\left(C-2\right)\times H\times W\times 3}$ . That is, every three adjacent spectral channels in **M** compose a three-channel image, and thus we will obtain (*C* − 2) images.

Afterwards, each three-channel image in the sequence is matched with labels for each category in the training set. Then, the matched labels are initialized as the point prompts of SAM. Following a category-wise processing manner, the current category label serves as the foreground, while the remaining category labels are considered as background for predicting the mask of the current category. Setting a confidence threshold, we designate labels with confidence scores above the threshold as foreground and continue to designate the remaining labels as background iteratively optimizing the generated labels. Taking point *a* as an example, the threshold is set as follows:


(1) $$y_{a}^{\left(i\right)}=1\;[p_{a}^{\left(i\right)}\geq \tau_{h}]-1\;\left[p_{a}^{\left(i\right)}\leq \tau_{l}\right],$$

where $y_{a}^{\left(i\right)}$ represents the predicted label for a in category i , and $p_{a}^{\left(i\right)}$ denotes the confidence score of the prediction. The parameters $\tau_{h}$ and $\tau_{l}$ are the confidence thresholds. According to the equation, a value of $y_{a}^{\left(i\right)}=1$ indicates the foreground, while $y_{a}^{\left(i\right)}=-1$ denotes the background.

### 2.2 Spectral voting

When splitting the hyperspectral image $M\in {\mathbb{R}}^{H\times W\times C}$ to $Q\in {\mathbb{R}}^{\left(C-2\right)\times H\times W\times 3}$ along the spectral dimension and cyclically predicting masks, category confusion may arise. To better leverage spectral information and mitigate this issue, we propose a spectral voting strategy. Assuming there are *K* categories of objects in the hyperspectral image, the prediction head of SAM generates an output tensor comprising (*C* − 2) × *K* masks. For each pixel *a* and category *i*, let *N* (*a,i*) denote the number of binary classification predictions corresponding to category *i*.

Specifically, if the prediction for the *j*th image in the current category is positive, *N* (*a,i*) increases by 1. Otherwise, it remains 0. Subsequently, compute the total number of predictions for that pixel across all categories *T*(*a*) by


(2) $$T\left(a\right)=\sum_{j=1}^{C-2}N_{i}\left(a,i\right).$$

After *k* rounds of processing, choose the category with the highest total prediction count as the final category for that pixel:


(3) $$\tilde{y}\left(a\right)=\text{arg }\underset{i}{\max}T\;(a,i),$$

where $\tilde{y}\left(a\right)$ means the final category for pixel *a*. By employing this process, each pixel undergoes (*C* − 2) predictions for every category and is ultimately assigned to the category with the highest total prediction count.

### 2.3 Spatial-information-consistency-driven loss function

To optimize the point prompts, we introduce a spatial-information-consistency-driven loss function. Specifically, in the context of the label generation process for the *i*th category, the feature set of the point prompts is represented as ***F_i_*** = {***f_i_*_1_** *,…,**f_ij_**,…,**f_in_***}. The feature of a sample *a* is denoted by *f_a_*, and the similarity *S_aj_* between *a* and a reference prompt point can be calculated as:


(4) $$S_{aj}=\frac{\langle f_{a},f_{ij}\rangle }{{\left\|f_{a}\right\|}_{2}{\left\|f_{ij}\right\|}_{2}},$$

where *S_aj_* represents the similarity between ***f_a_*** and ***f_ij_***, with ***f_ij_*** ∈ ***F_i_*** being the feature corresponding to the *i*th category label. The confidence $p_{a}^{\left(i\right)}$ of sample *a* is calculated as the average of its similarity with all labels, and is expressed as:


(5) $$p_{a}^{\left(i\right)}=\frac{1}{n}\sum_{j=0}^{n}S_{aj}.$$

During the training phase of the Prompt Encoder in SAM, we freeze most of the parameters of the feature decoder and update only the parameters of the first-layer decoder. A spatial-information-consistency-driven loss function is constructed by calculating the feature similarity between the samples of pseudo labels and those of ground-truth labels. This loss is then used to update the Prompt Encoder through backpropagation. In each iteration, IPG selects high-confidence pseudo labels as positive samples to optimize the generation of segmentation maps. The incoming “point prompts” are based on the most recently selected highconfidence pseudo labels, which are treated as new positive samples and simultaneously serve as prompts to SAM, ensuring continuous optimization and updating of the segmentation masks.

Specifically, the loss function for training the Prompt Encoder is designed to minimize the distance between samples with generated labels and those with labels from the same category, while maximizing the separation from other categories. To achieve this purpose, we employ a binary classification loss function to optimize the pseudo label generation framework:


(6) $${ℒ}_{\text{i}}=\sum_{a}g_{a}^{\left(i\right)}\left[\text{log }\left(p_{a}^{\left(i\right)}\right)+\sum_{j=1,j\neq i}^{K}\text{log }\left(1-p_{a}^{\left(j\right)}\right)\right],$$

where $g_{a}^{\left(i\right)}$ indicates whether pseudo labels are used for training the network, and can be calculated as:


(7) $$g_{a}^{\left(i\right)}=1\left[u\left(p_{a}^{\left(i\right)}\right)\leq \kappa_{h}\right]1\left[p_{a}^{\left(i\right)}\geq \tau_{h}\right],$$

where $u\left(p_{a}^{\left(i\right)}\right)$ quantifies the uncertainty of the pseudo labels and can be calculated using Equation 8. The parameter $\kappa_{h}$ represents the threshold for the uncertainty of positive samples. When the uncertainty falls below this threshold, indicating that the pseudo label is considered reliable, we set $p_{a}^{\left(i\right)}=1$ , thereby allowing the label to be used in subsequent classification task.


(8) $$u\left(p_{a}^{\left(i\right)}\right)=\sqrt{\frac{1}{n}\sum_{j=0}^{n}{\left(S_{aj}-p_{a}^{\left(i\right)}\right)}^{2}}.$$

### 2.4 IPG-based hyperspectral image classification

**Figure 3:**
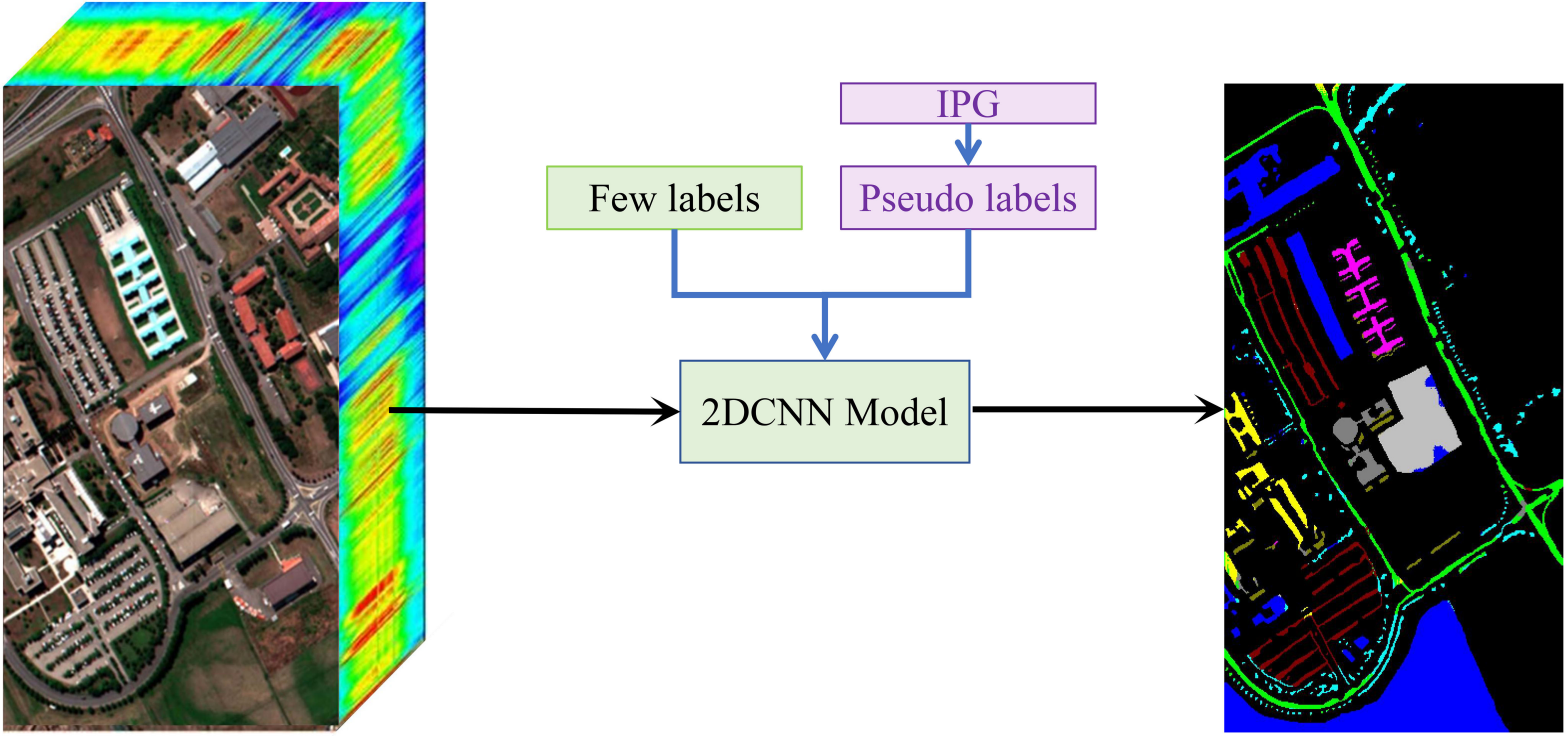
Illustration of the application of our IPG framework in an hyperspectral image classification network. Specifically, the pseudo labels generated by IPG are combined with the ground-truth labels to train the classification network, thereby improving its performance.

In hyperspectral image classification, the IPG framework plays a crucial role in enhancing the training process. After a sufficient number of iterations, the high-confidence pseudo labels generated by IPG are combined with the limited labeled samples, thereby enriching the training data for the 2D Convolutional Neural Network (2DCNN)-based classification model, as shown in **Figure 3**. This approach effectively utilizes both the scarce labeled data and the pseudo labels to enable more efficient feature learning. The learned features are then passed through the classification block of the model, which generates the final segmentation map by predicting category labels for each pixel in the hyperspectral image.

## 3 Experimental results and discussion

### 3.1 Experimental settings

To evaluate the efficacy of the proposed framework, we conducted experiments on two publicly available datasets: the Indian Pines dataset (Zare and Gader, 2008) and the Pavia University dataset (Huang and Zhang, 2009). During the training sample generation process, we employed a random sampling strategy. This approach ensures a diverse representation of samples, contributing to the robustness and generalizability of the model.

We compared our proposed method with several representative classification approaches, including Support Vector Machine (SVM) (Melgani and Bruzzone, 2004), Contextual Deep CNN (CDCNN) (Lee and Kwon, 2017), Spectral-Spatial Residual Network (SSRN) (Zhong et al., 2018), Double-Branch MultiAttention Mechanism Network (DBMA) (Ma et al., 2019), Adaptive Spectral-Spatial Kernel ResNet (A2S2K) (Roy et al., 2020), Discriminative Co-alignment (DCA) (Zhang et al., 2021), Dual-layer Deep Spatial Manifold Representation (SMR-EG) (Wang et al., 2022), and the Semi-Supervised Long-Tailed Learning Framework with Spatial Neighborhood Information (SLN-SNI) (Feng et al., 2023).

To ensure a comprehensive and fair comparison among the methods, we employed three additional metrics in addition to single-category classification accuracy: overall accuracy (OA) (Li et al., 2021), average accuracy (AA) (Li et al., 2022), and Kappa coefficient (Kappa) (Li et al., 2023, 2024). These metrics provide a more nuanced understanding of model performance by capturing different aspects of classification effectiveness. The calculations for these three metrics are detailed below:


(9) $$\text{OA}=\frac{\sum_{i=1}^{N}TP_{i}}{\sum_{i=1}^{N}Total_{i}},$$

(10) $$\text{AA}=\frac{1}{N}\sum_{i=1}^{N}\frac{TP_{i}}{Total_{i}},$$

(11) $$\text{Kappa}=\frac{\text{OA}-P_{e}}{1-P_{e}},$$

where *N* is the number of categories, *TP_i_* represents the number of true positives for the *i*th category, and *Total_i_* refers to the total number of samples in the *i*th category, including both correctly and incorrectly classified samples. Additionally, *P_e_* is calculated as follows:


(12) $$P_{e}=\frac{\sum_{i=1}^{N}\left[Total_{i}\times \left(TP_{i}+FP_{i}\right)\right]}{{\left(\sum_{i=1}^{N}Total_{i}\right)}^{2}},$$

where *FP_i_* represents the number of false positives for the *i*th category.

### 3.2 Implementation details

In our proposed method, we initialized an equal number of training samples, each of size 9 × 9, with five samples per category. Using SAM, we actively selected one sample per category per epoch for iterative optimization in each subsequent round. In the IPG model, we set the parameters as follows: *κ_h_* = 0.2, *τ_h_* = 0.8, and *τ_l_* = 0.5. Given that our framework operates iteratively, and based on the ablation results in Section 3.5, we set the number of iterations to 50 to balance efficiency and effectiveness. After several rounds of iterative optimization, the generated pseudo samples were combined with ground-truth samples to train the 2DCNN, aiming for high precision. The network was trained using an SGD optimizer with the following default parameters: learning rate = 0.05, momentum = 0.7, and weight decay = 0.0001. All implementations were based on the PyTorch backend and executed on a desktop equipped with a single NVIDIA A100 GPU.

Throughout the experiments, we ensured consistency with the control method in terms of training samples, test samples, and learning rate, thus establishing a fair comparison between all models evaluated.

### 3.3 Qualitative evaluation results

**Figure 4:**
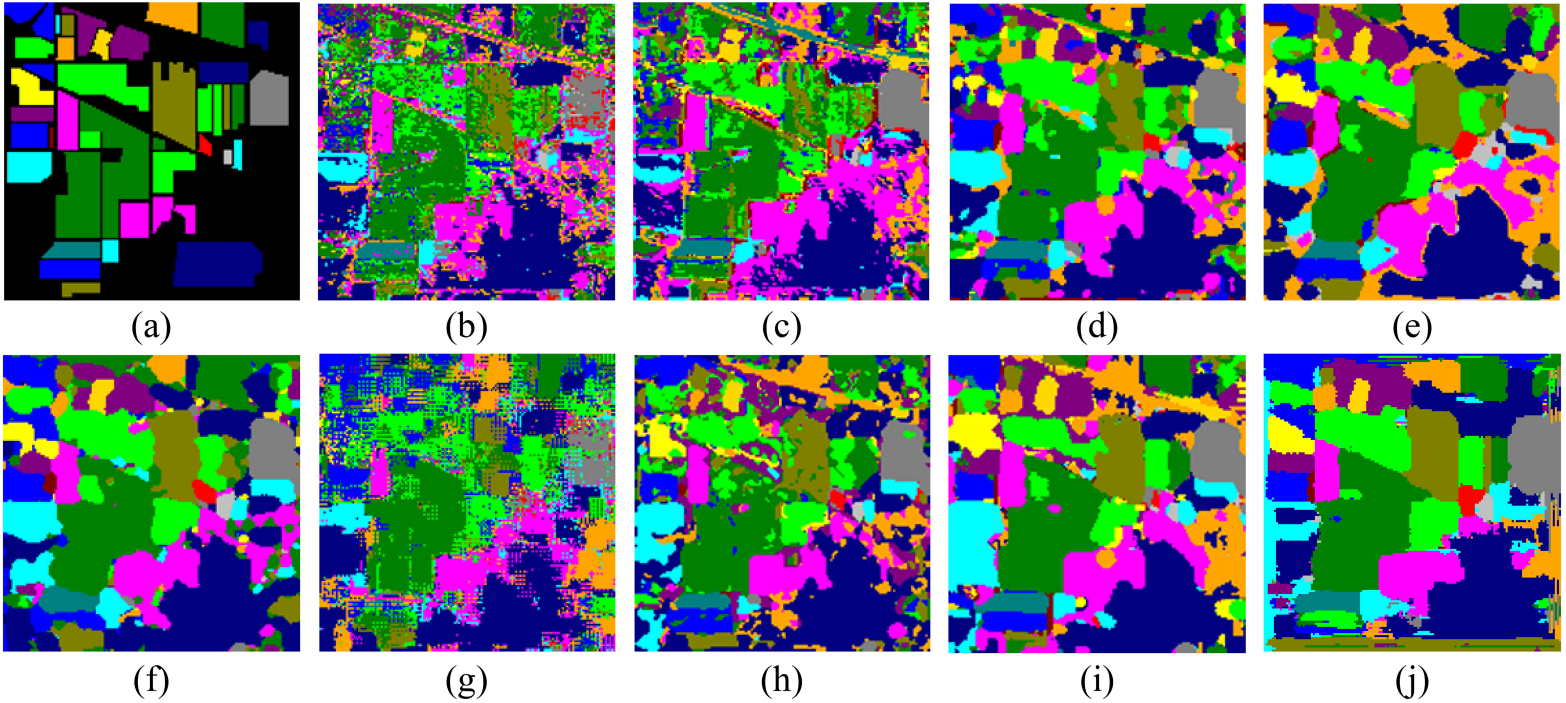
Visual comparison of classification results on the Indian Pines dataset. **(A)** Ground-truth map, **(B)** SVM, **(C)** CDCNN, **(D)** SSRN, **(E)** DBMA, **(F)** A2S2K, **(G)** DCA, **(H)** SMR-EG, **(I)** SLN-SNI, and **(J)** Ours.

**Figure 4** displays the classification map generated by various methods on the Indian Pines dataset, providing a comprehensive assessment of model performance. It is clear that our method significantly outperforms competing algorithms in terms of visual quality, showcasing superior integrity in overall segmentation. The classification map produced by our proposed method exhibits sharper boundaries, reduced noise, and more distinct category separations, thereby enhancing the interpretability and reliability of hyperspectral image classification.

**Figure 5:**
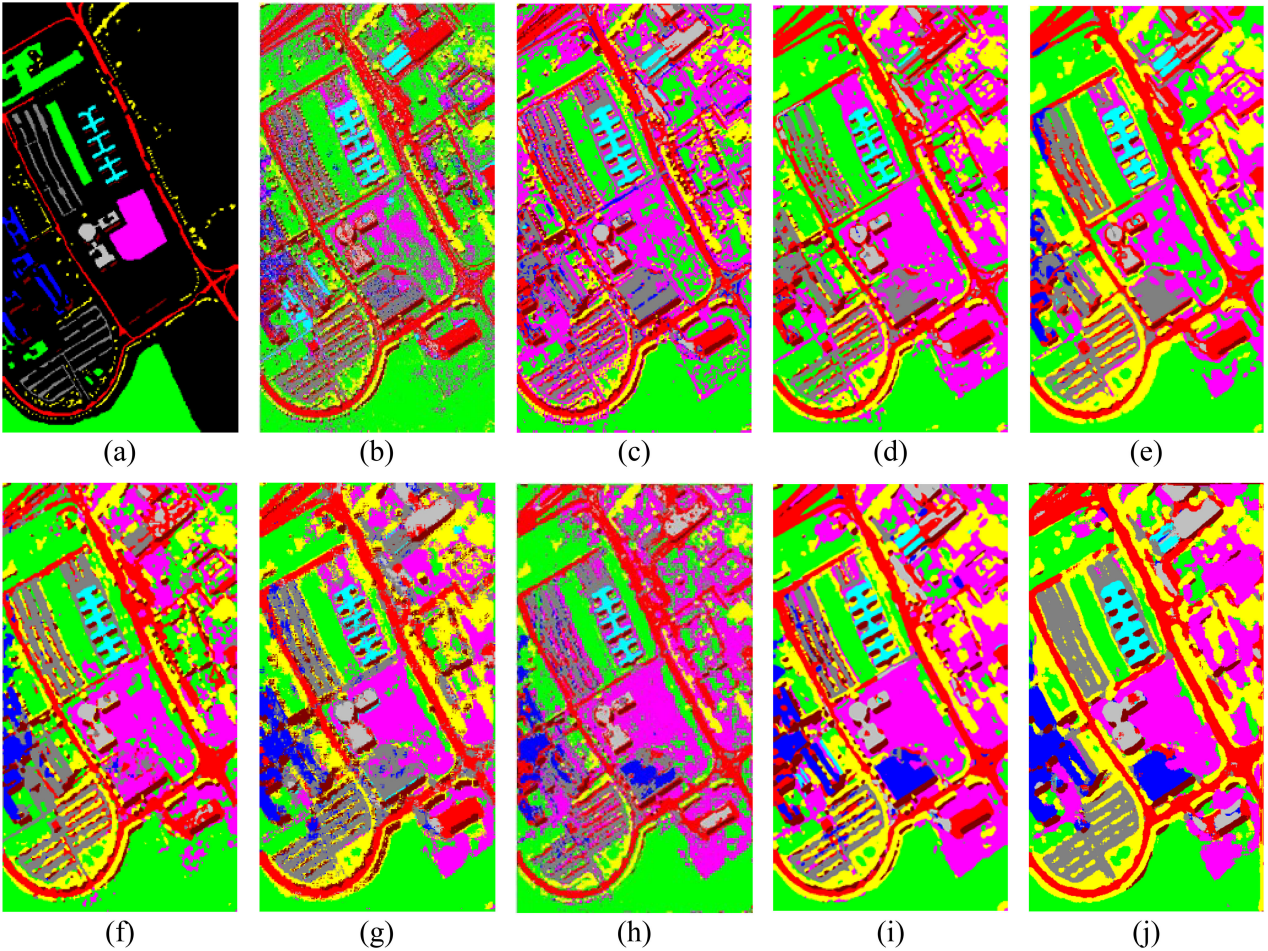
Visual comparison of classification results on the Pavia University dataset. **(A)** Ground-truth map, **(B)** SVM, **(C)** CDCNN, **(D)** SSRN, **(E)** DBMA, **(F)** A2S2K, **(G)** DCA, **(H)** SMR-EG, **(I)** SLN-SNI, and **(J)** Ours.

In contrast, **Figure 5** presents the classification results of the comparison methods on the Pavia University dataset. Especially in regions corresponding to categories such as Asphalt and Trees, the classification results are clear with well-defined boundaries, and there are fewer classification errors. In contrast, traditional methods like SVM and CDCNN exhibit significant noise in certain land cover areas, particularly in complex regions such as Buildings, where the classification results are more disordered. In comparison, our method not only effectively reduces noise but also demonstrates more accurate boundary recognition.

### 3.4 Quantitative evaluation results

**Tables 1** and **2** summarize the overall accuracy (OA), average accuracy (AA), and Kappa coefficient (Kappa) values for various methods evaluated on two real datasets. The results indicate that our proposed method achieves substantial improvements over comparative techniques across all evaluation metrics, demonstrating its robustness and effectiveness. The detailed results and discussions are presented below.

**Table 1:** Classification performance obtained by different methods on the Indian Pines dataset.

Category	SVM	CDCNN	SSRN	DBMA	A2S2K	DCA	SMR-EG	SLN-SNI	Ours
1	18.69	29.79	66.67	78.00	80.00	39.13	50.00	39.13	**100.00**
2	53.16	69.88	78.42	85.85	90.45	91.46	61.69	91.74	**95.26**
3	48.57	48.48	80.71	86.68	90.68	72.53	32.47	**94.22**	74.82
4	34.48	44.44	95.74	77.02	**97.56**	8.86	82.05	92.83	92.74
5	89.97	88.43	**98.80**	86.14	96.56	38.51	88.18	92.55	74.65
6	78.93	86.59	95.86	97.76	91.41	77.26	86.49	**98.36**	97.46
7	45.10	35.29	92.00	38.00	**100.00**	3.57	42.86	**100.00**	**100.00**
8	89.47	88.63	97.65	97.85	97.22	99.37	84.85	99.79	**100.00**
9	24.14	35.42	64.29	28.89	58.82	0.00	0.00	**100.00**	**100.00**
10	63.91	49.29	81.73	81.50	86.12	91.05	56.21	86.73	**96.07**
11	58.23	62.69	84.50	91.76	85.54	77.47	67.43	93.56	**97.69**
12	43.44	36.16	92.33	78.32	90.24	4.86	30.31	81.11	**93.81**
13	88.52	77.07	**100.00**	99.39	94.47	0.00	89.47	**100.00**	**100.00**
14	85.60	73.97	92.51	92.87	94.06	71.78	85.49	97.79	**99.92**
15	57.59	77.48	88.47	88.92	**99.54**	20.21	72.60	79.53	76.05
16	98.44	75.68	98.86	68.81	87.91	87.10	94.29	**100.00**	92.63
OA (%)	65.02	65.10	86.93	88.89	90.16	68.85	66.31	92.53	**93.51**
AA (%)	61.77	61.21	88.03	82.36	90.04	48.94	64.03	90.46	**93.19**
Kappa (%)	59.43	59.88	85.01	87.32	88.70	64.28	61.11	91.48	**92.58**

Optimal results are bolded.

**Table 2:** Classification performance obtained by different methods on the Pavia University dataset.

Category	SVM	CDCNN	SSRN	DBMA	A2S2K	DCA	SMR-EG	SLN-SNI	Ours
1	83.46	87.99	94.50	93.56	78.57	76.64	98.28	95.76	**99.67**
2	88.17	94.03	98.70	96.98	94.55	99.14	98.86	99.71	**99.77**
3	72.49	95.64	**99.54**	98.89	84.61	57.27	82.23	94.62	97.34
4	95.55	94.59	98.34	97.93	99.43	67.20	96.12	97.75	**99.87**
5	90.65	99.69	99.25	99.33	**100.00**	45.20	99.84	**100.00**	**100.00**
6	76.37	83.20	91.47	97.40	95.49	67.65	96.31	**99.94**	98.73
7	69.24	97.07	**99.73**	96.05	95.14	39.25	80.57	99.55	97.97
8	72.12	64.57	79.08	85.26	76.59	99.21	76.92	95.11	**99.73**
9	99.89	96.59	99.79	**100.00**	96.98	88.91	79.74	**100.00**	99.79
OA (%)	84.43	87.18	95.07	95.56	90.44	83.83	94.12	98.35	**99.46**
AA (%)	83.10	83.71	95.60	96.16	91.26	71.16	89.99	98.05	**99.20**
Kappa (%)	79.05	82.92	93.47	94.06	87.14	78.23	92.23	97.81	**99.25**

Optimal results are bolded.

In **Table 1**, our method attains 100% accuracy in predicting five categories, with only three categories showing accuracy below 90%. This highlights the model’s robustness in handling diverse categories. However, performance of our method in the third and fifth categories is comparatively lower, likely due to the similar spectral signatures of these categories, which complicates their distinction. The semi-supervised nature of our model, though powerful, may struggle to fully capture the nuances of these categories, especially as the iterative pseudo-labeling process can introduce noisy labels that degrade subsequent classification accuracy.

Further supporting this, **Table 2** demonstrates that our approach significantly outperforms previous models in two specific categories, underscoring the framework’s capability to enhance classification accuracy in hyperspectral image analysis. These quantitative findings validate the effectiveness of our method in advancing hyperspectral classification performance across various challenging scenarios.

### 3.5 Ablation study

#### 3.5.1 Ablation of different module combinations

**Table 3** presents the contributions of various modules within our proposed method to classification accuracy. In this table, “SAM” denotes the framework based on the Segment Anything Model, while “SCC” refers to the spatial-information-consistency-driven loss function, and “SV” indicates the spectral voting strategy. If Spectral Voting strategy absent, it indicates that hyperspectral images are reduced to three-channel images for processing. The results demonstrate that incorporating the IPG framework significantly improves classification accuracy. This improvement can be attributed to the use of unsupervised large models in the semi-supervised generation of pseudo labels. Additionally, the spatial-informationconsistency-driven loss function and the spectral voting strategy further enhance classification accuracy within the IPG framework.

**Table 3:** Ablation study of different module combinations on the Pavia University dataset.

SAM	SCC	SV	OA (%)	AA (%)	Kappa (%)
×	×	×	93.15	94.03	92.16
✓	×	×	96.19	95.83	95.59
✓	✓	×	97.83	97.08	96.72
✓	✓	✓	**99.46**	**99.20**	**99.25**

Optimal results are bolded.

The symbols ✓ and × indicate the classification model with or without the corresponding module, respectively.

#### 3.5.2 Ablation for different iteration numbers in spatial-information-consistency-driven loss function

To evaluate the effect of iteration numbers in the spatial-information-consistency-driven loss function on classification performance, we conducted experiments using the Pavia University dataset. As shown in **Table 4**, increasing the number of iterations initially results in a consistent improvement in classification accuracy, with only a slight increase in running time. However, when the iteration number surpasses 50, accuracy begins to decline, and the running time increases sharply. This can be attributed to the increasing number of pseudo labels fed into SAM in each iteration, leading to increased inference time. Meanwhile, as the number of iterations grows, the consistency-constrained pseudo labels tend to converge too closely to the labeled data, reducing the inclusion of useful spatial information and ultimately causing a drop in accuracy. These ablation results demonstrate the importance of selecting an optimal number of iterations to balance classification accuracy and computational efficiency.

**Table 4:** Ablation study of different iteration numbers in spatialinformation-consistency-driven loss function on the Pavia University dataset.

Epoch of iterations	10	30	50	70	90
OA (%)	97.79	98.43	**99.46**	98.26	96.82
AA (%)	97.34	97.87	**99.20**	97.12	96.87
Kappa (%)	97.14	98.15	**99.25**	97.78	96.84
Time (Seconds)	**58.95**	234.07	537.82	958.79	1540.68

Optimal results are bolded.

## 4 Conclusion

This paper introduces an iterative pseudo label generation (IPG) framework for hyperspectral image classification. The proposed approach integrates the Segment Anything Model (SAM) with a spectral voting strategy, effectively leveraging the rich spectral information in hyperspectral images for label estimation. Experimental results confirm that the IPG framework significantly improves classification performance, even with limited annotations. Despite its promising results, this study has some limitations. First, the performance of the IPG framework can be affected by the method used to group spectral bands, which may result in variability in outcomes across different datasets. Second, the model’s dependence on the quality of initial annotated labels may restrict its effectiveness in scenarios with insufficient or low-quality labeled data. In future work, we will further enhance the proposed method and evaluate its performance on a broader range of datasets to reinforce its robustness and demonstrate its generalizability.

## Data Availability

The datasets used in this study is available at: https://www.ehu.eus/ccwintco/index.php/Hyperspectral_Remote_Sensing_Scenes.
